# Chronic obstructive pulmonary disease and lung cancer: access to palliative care, emergency room visits and hospital deaths

**DOI:** 10.1186/s12890-021-01533-3

**Published:** 2021-05-19

**Authors:** Peter Strang, Per Fürst, Christel Hedman, Jenny Bergqvist, Helena Adlitzer, Torbjörn Schultz

**Affiliations:** 1 Department of Oncology-Pathology, Karolinska Institutet, Regional Cancer Centre in Stockholm, Gotland, Sweden; 2 R & D Department, Stockholms Sjukhem Foundation, P.O. Box 12230, 102 26 Stockholm, Sweden; 3 Department of Oncology-Pathology, Karolinska Institutet, Stockholm, Sweden; 4 Department of Molecular Medicine and Surgery, Karolinska Institutet, Stockholm, Sweden; 5 Department of Medical Epidemiology and Biostatistics, Karolinska Institutet, Stockholm, Sweden; 6 Department of Surgery, Capio St Görans Sjukhus, Stockholm, Sweden; 7 Regional Cancer Centre in Stockholm, Gotland, Sweden

**Keywords:** Chronic obstructive pulmonary disease, Lung cancer, Palliative care services, Place of death

## Abstract

**Background:**

Despite the severe symptoms experienced by dying COPD patients, specialized palliative care (SPC) services focus mainly on cancer patients. We aimed to study the access to SPC that COPD and lung cancer (LC) patients receive and how that access affects the need for acute hospital care.

**Methods:**

A descriptive regional registry study using data acquired through VAL, the Stockholm Regional Council’s central data warehouse, which covers nearly all healthcare use in the county of Stockholm. All the patients who died of COPD or LC from 2015 to 2019 were included. T-tests, chi-2 tests, and univariable and multivariable logistic regression analyses were performed on the accumulated data.

**Results:**

In total, 6479 patients, (2917 with COPD and 3562 with LC) were studied. The patients with LC had more access to SPC during the last three months of life than did those with COPD (77% vs. 18%, respectively; p < .0001), whereas patients with COPD were more likely to be residents of nursing homes than those with LC (32% vs. 9%, respectively; p < .0001). Higher socioeconomic status (SES) (p < .01) and patient age < 80 years (p < .001) were associated with increased access to SPC for LC patients. Access to SPC correlated with fewer emergency room visits (p < .0001 for both COPD and LC patients) and fewer admissions to acute hospitals during the last month of life (p < .0001 for both groups). More COPD patients died in acute hospitals than lung cancer patients, (39% vs. 20%; χ^2^ = 287, p < .0001), with significantly lower figures for those who had access to SPC (p < .0001).

**Conclusions:**

Compared to dying COPD patients, LC patients have more access to SPC. Access to SPC reduces the need for emergency room visits and admissions to acute hospitals.

## Background

Although mild forms of chronic obstructive pulmonary disease (COPD) may remain undiagnosed, severe forms cause distressing and complex symptoms, especially in the end-of-life (EOL) [1]. While respiratory symptoms, such as cough, sputum production, and dyspnea can be disturbing for the patient early in the course of the disease, later on, comorbidities, such as ischemic heart disease, heart failure and arrythmias, thromboses, lung embolisms, osteoporosis, and lung cancer (LC) may constitute a clinical burden [1]. As concluded in a review, comorbidities actually account for two-thirds of the deaths of COPD patients, whereas respiratory failure is the main cause in the others [2].

Today, COPD is a major cause of death in Sweden, with almost 3000 deaths annually, compared to approximately 3500 deaths from LC [3]. Despite successive deterioration, the typical COPD trajectory is an illness with stable periods, abrupted by exacerbations, often accompanied by infections but also by concomitant heart failure [1, 4]. The severe exacerbations may end in recovery or, conversely, death. For this reason, prognostication is difficult. Efforts have been made to establish reliable prognostic factors, both in the form of single factors and as prognostic indices, with some consensus on group level; however, difficulties in establishing a prognosis for individual patients remain [4, 5]. Respiratory factors, such as dyspnea, FEV1%, COPD exacerbations, exercise capacity, inspiratory fraction and inspiratory capacity are frequently suggested as predictors of death, along with comorbidities and older age [6]. To improve the predictive capacity of these variables, constructs have been used, such as BODE, comprising Body Mass Index, Obstruction, Dyspnea, and Exercise capacity [7], ADO (Age, Dyspnea, and Obstruction) [8, 9] and DOSE (Dyspnea, Obstruction, Smoking, and Exercise capacity) [10].

Nevertheless, strong single factors such as dyspnea [6] and composites, such as BODE, DOSE, and ADO mainly predict survival prospects at the group level, but to a lesser degree in individual cases [5, 6, 11, 12]. For this reason, even experienced pulmonary specialists in Sweden are reluctant to refer severely ill COPD patients for specialized palliative care (SPC) services, although, they do not hesitate to refer LC patients with a fairly predictable disease trajectory [13]. This phenomenon is not isolated to Sweden, as similar observations have been made internationally, as shown in a recent systematic review [4]. Consequently, COPD is more often seen as a chronic disease, even in EOL situations. Therefore, COPD patients are more likely than LC patients to receive life-sustaining measures, such as non-invasive and invasive ventilation and cardiopulmonary resuscitation [4, 14, 15].

In Sweden, efforts have been made to increase awareness about the benefits of a palliative care approach in EOL situations, regardless of the place of care. Therefore, general palliative care is consistently offered in all healthcare facilities, including nursing homes. SPC is offered for those with complex symptoms and greater needs, mainly in the form of advanced palliative home care or hospital palliative care units. Both types of care are staffed 24 h a day, 7 days a week with physicians, registered nurses, physiotherapists, occupational therapists, dieticians, assistant nurses, and other medical professionals [16].

Considering the effectiveness of SPC for symptom control and generalized support [16] and the documented positive outcomes for patients with COPD [17], it is reasonable to assume that both LC and COPD patients would be candidates for SPC. Butler et al. recently reviewed comparative studies on COPD and LC [4], however, 10 of the 20 studies reviewed were based on data collected more than 10 years earlier and, among the more recent studies, only two included 1000 or more patients [18, 19]. For this reason, we aimed to conduct a large study using recent data, as inclusion criteria for admission to palliative care services for non-malignant conditions such as COPD are changing rapidly.

### Aims

In this study, we aimed to retrospectively compare the access to SPC of COPD and LC patients during their last three months of life and analyze to what extent age, sex, or socioeconomic status (SES) influences the opportunities to receive SPC. A further aim was to study whether access to SPC reduced acute emergency visits and admissions to acute hospitals during the last month of life.

## Patients and methods

The Methods and Results sections are, when possible, reported based on the Strengthening the Reporting of Observational Studies in Epidemiology (STROBE) criteria [20].

### Study design

We conducted a descriptive regional registry data study using VAL, the Stockholm Region's central data warehouse. There are separate registers within VAL for outpatient visits to hospitals and hospital stays. Complete data were retrieved for patients who died between 2015 and 2019, and various aspects of healthcare consumption were compared between those who died from either COPD or LC. For each patient included in the analysis, data were collected for the 12 months preceding the date of death. The data were further analyzed according to age, sex, living arrangements (residents in nursing homes versus all others), and SES using Mosaic [21–23]. Mosaic provides socioeconomic information and allows the county council (Stockholm Region) to define and allocate different areas of residence within the county of Stockholm to one of three different socioeconomic classes (Mosaic 1–3), mainly based on income and education, but also factoring in other elements, such as cultural aspects, lifestyle, and living arrangements. The county of Stockholm is divided into 1300 small areas, and each area is classified as Mosaic 1, 2, or 3. The three groups are approximately equal in size, but Mosaic group 1 is the most affluent.

### Population

All the patients over the age of 18 who died during the years 2015 to 2019 with a main diagnosis of COPD (J 44 in ICD-10) or LC (C34) were included in this study.

### Variables

Access to SPC and the need for emergency room visits and/or admission to acute hospitals were used as outcome measures. Age, sex, living arrangements (nursing homes versus all others), and Mosaic groups as a measure of SES were used as explanatory variables.

### Selection bias

*Dropouts*: As reporting data to VAL is an obligation for each clinic/care unit in the region, the data are complete, with few missing values. This means that any individual who has used public healthcare during a given year is included in the VAL databases; this also applies to most forms of private care, as private healthcare suppliers have economic agreements with the regional council.

*Nursing home residents*: Nursing home residents were identified through registrations of medical interventions by physicians, as such care is exclusive to nursing home residents and has a unique, identifiable code. It is unlikely that a nursing home resident does not have a single registration during their last year of life. If so, they were not included in the analysis.

### Study size

This study covered all cohorts, i.e., all deaths (all causes) during the years 2015–2019. Therefore, no power calculations were performed.

### Statistical methods, missing data

T-tests and chi-2 tests were used to compare the proportions. The few missing data were not substituted. Univariable logistic regression analyses were performed and variables with a p-value of < 0.20 were then entered in multivariable logistic regression models. The SAS version 9.4 was used for statistics.

## Results

During the years 2015–2019, there were 2917 deaths from COPD and 3562 deaths from LC, for a total of 6489 patients. The patients who died from COPD were older (80.2 years) than those with LC (72.7 years) (p < 0.0001). The proportions of deaths in different age groups are shown in Table 1. A larger proportion of COPD than LC patients were female, (59% vs. 52.4% χ^2^ = 28.9, p < 0.0001). During the last 3 months of life, 17.7% of the patients with COPD and 76.7% of the patients with LC had access to specialized palliative care (χ^2^ = 2235, p < 0.0001) (Table 1).

**Table 1:** Characteristics and care utilization for 6479 patients who died in COPD (n = 2917) or in LC (n = 3562)

Characteristics and care utilization	COPD	LC	p-value
*Deaths*	2917	3562	
*Age*, all years (sd)	80.2 (9.1)	72.7 (9.9)	< .0001
Women	80.9 (9.0)	72.9 (10.0)	< .0001
Men	79.3 (9.0)	72.6 (9.9)	< .0001
*Age groups*			
20–39 years, n (%)	0 (0.0)	9 (0.3)	< .01
40–59 years, n (%)	58 (2.0)	313 (8.8)	< .0001
60–69 years, n (%)	293 (10.0)	859 (24.1)	< .0001
70–79 years, n (%)	928 (31.8)	1522 (42.7)	< .0001
80 years or older, n (%)	1638 (56.2)	859 (24.1)	< .0001
*Sex—women (%)*	59.0	52.4	< .0001
*Access to SPC (%)*	17.7	76.7	< .0001
*Care in nursing homes (%)*	31.5	9.4	< .0001
Age, nursing home residents (years)	84	79	< .0001
*Emergency room visits, all (%)*	64.4	45.1	< .0001
Patients with access to SPC (%)	52^2^	39^1^	
Patients without access to SPC (%)	67^2^	66^1^	
*Hospital as place of death (%)*	39	20	< .0001
Patients with access to SPC (%)	11.8^2^	8.6^1^	
Patients without access to SPC (%)	45.2^2^	58.0^1^	

T-test was used for comparison of age. Chi-2 test was used for comparison of proportions

^1^The difference between those LC patients with and without access to SPC (vertical comparison in the Table) was significant (p < .0001) both as regards emergency room visits and hospital as a place of death

^2^The difference between those COPD patients with and without access to SPC (vertical comparison in the Table) was significant (p < .0001) both as regards emergency room visits and hospital as a place of death

### Univariable logistic regression (both groups)

In the initial univariable analyses, the likelihood of being admitted to specialized palliative care services during the last 3 months of life was significantly higher for men than women, for persons belonging to higher socioeconomic classes (Mosaic 1 and 2), and for younger patients. The biggest difference was related to diagnosis, patients with LC were much more likely to have access to specialized palliative care than those with COPD (Table 2).

**Table 2:** Access to SPC. Odds ratio (OR) for different variables

Variable	Univariable analysis		Multivariable analysis	
	OR (95% CI) p-value	p-value	OR (95% CI) p-value	p-value
*Sex*				
Women	0.90 (0.82–.995)	.04	1.10 (0.97–1.24)	.14 (ns)
Men	Ref		Ref	
*Socio-economic status*				
Mosaic group 1	1.32 (1.16–1.50)	< .0001	1.33 (1.14–1.56)	.0004
Mosaic group 2	1.26 (1.13–1.41)	< .0001	1.16 (1.01–1.33) .04	.04
Mosaic group 3	Ref		Ref	
*Age groups*				
18–39 years	14.64 (1.83–116.94)	.01	3.09 (0.38–24.79)	.29 (ns)
40–59 years	3.98 (3.15–5.03)	< .0001	1.38 (1.05–1.82)	.02
60–79 years	2.57 (2.32–2.86)	< .0001	1.37 (1.19–1.56)	< .0001
80 years or older	Ref		Ref	
*Diagnosis*				
COPD	0.06 (0.06–0.07)	< .0001	0.07 (0.06–0.08)	< .0001
Lung cancer	Ref		Ref	

### Multivariable logistic regression (both groups)

We tested three different models for the likelihood of being admitted to specialized palliative care services during the last three months of life, with similar results. Diagnosis (COPD and LC), sex, and SES (Mosaic 1–3) were included in the first model. In the second model, age was entered as a continuous variable, whereas age was included as age categories (18–39, 40–59, 60–79, and ≥ 80 years) in the third model. The third model is shown in Table 2. Sex differences were attenuated in all three models. The odds ratio for women was 0.90 and significant in the univariable model; however, it was 1.1, but not significant, in the multivariable model.

### COPD (separate analysis)

In a separate univariable analysis of COPD patients, neither sex nor SES (Mosaic groups) was significantly associated with the probability of receiving specialized palliative care, whereas there was an association with age groups; i.e., patients aged 70–79 years had an odds ratio of 1.31, compared to the patients aged ≥ 80 years (p = 0.01) (Table 3). The findings remained unchanged in the multivariable model that included the same explanatory variables (Table 3).

**Table 3:** COPD (only): Access to SPC. Odds ratio (OR) for different variables

Variable	Univariable analysis		Multivariable analysis	
	OR (95% CI)	p-value	OR (95% CI)	p-value
*Sex*				
Women	1.02 (0.84–1.24)	.85 (ns)	1.01 (0.83–1.22)	.94 (ns)
Men	Ref		Ref	
*Socio-economic status*				
Mosaic group 1	1.21 (0.95–1.54)	< .13 (ns)	1.19 (0.93–1.52)	.16 (ns)
Mosaic group 2	0.998 (0.80–1.24)	< .98 (ns)	1.00 (0.80–1.24)	.97 (ns)
Mosaic group 3	Ref		Ref	
*Age groups*				
40–59 years	0.36 (0.13–1.01))	.05 (ns)	0.38 (0.13–1.05)	.06 (ns)
60–69 years	0.75 (0.53–1.08)	.13 (ns)	0.79 (0.55–1.13)	.19 (ns)
70–79 years	1.31 (1.01–1.60)	.01	1.31 (1.07–1.61)	< .001
80 years or older	Ref		Ref	

### Lung cancer (separate analysis)

In contrast to the COPD patients, the LC patients from more affluent Mosaic groups were more likely to be admitted to specialized palliative care, as were patients from all the age groups between 40–79 years, compared with the oldest group (≥ 80 years old) (Table 4).

**Table 4:** Lung cancer (only): Access to SPC. Odds ratio (OR) for different variables

Variable	Univariable analysis		Multivariable analysis	
	OR (95% CI)	p-value	OR (95% CI)	p-value
*Sex*				
Women	1.14 (0.97–1.32)	.11 (ns)	1.14 (0.98–1.34)	.10 (ns)
Men	Ref		Ref	
*Socio-economic status*				
Mosaic group 1	1.39 (1.13–1.72)	.002	1.40 (1.13–1.73)	.002
Mosaic group 2	1.27 (1.06–1.51)	.009	1.25 (1.05–1.49)	.01
Mosaic group 3	Ref		Ref	
*Age groups*				
18–39 years	3.38 (0.42–27.14)	.25 (ns)	3.49 (0.43–28.16)	.24 (ns)
40–59 years	1.68 (1.22–2.29)	.001	1.78 (1.29–2.45)	.0004
60–69 years	1.48 (1.19–1.84)	.0004	1.49 (1.19–1.85)	.0004
70–79 years	1.58 (1.30–1.91)	< .0001	1.59 (1.31–1.93)	< .0001
80 years or older	Ref		Ref	

### Care in nursing homes

In total, 31.5% of the dying COPD patients, but only 9.4% of the dying LC patients (χ^2^ = 498, p < 0.0001), were cared for in nursing homes during the EOL. The nursing home residents with COPD were older than those with LC, (84 years vs. 79 years, respectively; p < 0.0001) (Table 1).

### Emergency room visits during the last month of life

During the last month of life, 64.4% and 45.1% of the patients with COPD and LC, respectively, at least one emergency room visit (χ^2^ = 240.2, p < 0.0001). The figure was 67% among the COPD patients without access to SPC, but the percentage was significantly lower for those with access (52%; χ^2^ = 48.2, p < 0.0001). The corresponding figures for the LC patients with and without access to SPC were 66% and 39%, respectively (χ^2^ = 183.9, p < 0.0001) (Table 1).

### Admissions to acute hospitals during the last month of life

The figures for admissions to acute hospitals during the last month of life were similar to those of emergency room visits. In total, 62% and 52% of the COPD and LC patients, respectively, were admitted to acute hospitals (χ^2^ = 52.9, p < 0.0001). For the COPD patients, 52% with access to palliative care were admitted to acute hospitals, whereas the figure was 64% for those without palliative care (χ^2^ = 25.6, p < 0.0001). For the LC patients, the figures were 48% with and 69% without access to SPC, respectively (χ^2^ = 116, p < 0.0001) (data not shown).

### Hospitals as the place of death

In total, more COPD patients than LC patients died in acute hospitals (39% vs. 20%, respectively; χ^2^ = 287, p < 0.0001). Those patients who had access to SPC died less frequently in acute hospitals than those without access (11.8% vs. 45.2% for COPD patients; χ^2^ = 198, p < 0.0001 and 8.6% vs. 58.0% for LC patients; χ^2^ = 965, p < 0.0001) (Table 1).

## Discussion

In summary, deaths from COPD and LC were more frequently seen for women than men, and those dying of COPD were older than those dying of LC. Access to SPC was more likely for persons with LC, and it was partially affected by age and SES, although not by sex. Access to SPC significantly reduced the need for emergency room visits, admissions to acute hospitals, and hospital deaths.

Although smoking was much more common among Swedish men in the 1950s and 1960s, tobacco consumption increased among female Swedes in the 1970s, in parallel with a reduced use in men. As the time lag between exposure and a diagnosed illness may be a question of decades, changes in the illness panorama also have a delay. In the 1970s, women had a much lower risk of dying from LC (approximately 10–15 deaths/100,000 compared to 40–55 deaths/100,000 for men) [24]. Since then, female deaths have increased, and male deaths have decreased. Today, both groups have incidences between 30 and 40/100,000 and, as shown in our data, deaths from LC are now somewhat more common among females, at least in the Stockholm region.

Equal access to SPC for patients with similar needs, regardless of sex, age, or SES, is a common goal in healthcare. However, inequalities still exist. The likelihood of receiving hospice care differs depending on the healthcare system. In a US Medicare study, cancer patients who were younger, male, black, unmarried, and those with lower incomes were less likely to receive hospice care [25]. In a recent review, higher SES and being female were variables associated with access to palliative care [26].

In this study, we found that access to palliative care was not related to sex, in contrast to a recent study on COPD and LC by Kendzerska et al., who observed that women received more palliative home care than men [19]. Moreover, for the COPD patients in our study, access to SPC was not correlated with SES, according to the Mosaic groups, but was so for the patients with LC; this was also the case in the multivariable regression models that included age and sex. The reason for this remains unclear. Similarly, age was a less significant factor for the COPD patients, but it was of great significance to those with LC; i.e., the oldest patients (≥ 80 years) had much less access to SPC, which is in line with previous studies [26].

Access to SPC has been associated with better symptom control and social support [17]. Obviously, SPC services are successful in the management of symptoms and acute conditions, which was indirectly reflected in this study; i.e., patients who were enrolled in SPC services made significantly fewer emergency room visits and were admitted to acute hospitals to a lesser extent during the last month of life than those not enrolled in SPC services, which is in good agreement with the study by Kendzerska et al. [19]. Moreover, only 12% of the patients with COPD and 9% of those with LC who had access to SPC died at acute hospitals, compared to 45% and 58%, respectively, of those who did not have access, implying that SPC unburdens acute hospital care. This is a question of quality, as many patients prefer home as their place of death [27].

Finally, neither all cancer nor COPD patients are in need of specialized palliative care at the end of life, although most of them would probably benefit from an earlier integration of palliative care with the disease-specific treatments, which was shown in a seminal randomized study by Temel et al., in newly diagnosed, metastatic non-small-cell lung cancer (NSCLC) [28]. Those who were randomized to palliative care interventions, in parallel with their cancer-specific treatment showed improved quality of life, improved mood and even an improved overall survival. Patients with far advanced COPD also have a heavy symptom burden [1] and should be candidates for palliative care interventions earlier than today. There are guidelines to facilitate earlier identification of COPD patients to support earlier recognition of patients nearing the end of life [29].

Access to palliative care for an individual patient depends both on the availability of services and on actually being referred. The latter aspect is important when comparing lung cancer and COPD patients: while pulmonary specialists have routines for referring lung cancer patients, they are more hesitant to refer COPD patients [13], due to the more unpredictable trajectory in COPD patients, who more often die from acute events after a stable period.”

### Strengths and limitations

As all healthcare in Sweden, with few exceptions, is financed by taxes, and reporting to the VAL databases is mandatory, the data have very few missing values.

A possible limitation to this study is that the diagnosis for each patient was not based on the death certificate, but on the primary diagnosis during the last episode of care, which, in the case of LC, was often strengthened by a diagnosis of secondary tumors (metastases). Therefore, we cannot exclude the possibility that the immediate cause of death for some of the patients in this study may have been something other than COPD or LC, such as cardiac arrest.

## Conclusions

Access to SPC is greater for patients with LC than for patients with COPD. Access is equal and fair in relation to sex, but for LC patients, access is associated with SES and age. SPC reduces the need for emergency visits and acute hospital care during the last month of life.

## Data Availability

The datasets generated, used and analyzed during the current study available from the corresponding author on reasonable request.

## Abbreviations

EOL: End-of-life
OR: Odds ratio
SES: Socioeconomic status
